# A blood-brain barrier model based on flexible tubes to tailor the biophysical and chemical environment for drug delivery testing

**DOI:** 10.1016/j.mtbio.2026.103307

**Published:** 2026-06-02

**Authors:** Maria Alexaki, Attilio Marino, Marie Celine Lefevre, Claudio Canale, Davide Odino, João F. Mano, Mariana B. Oliveira, Gianni Ciofani

**Affiliations:** a Smart Bio-Interfaces, Istituto Italiano di Tecnologia, Pontedera, Italy; b The Biorobotics Institute, Scuola Superiore Sant’Anna, Pontedera, Italy; c Department of Chemistry, CICECO-Aveiro Institute of Materials, University of Aveiro, Aveiro, Portugal; d Physics Department, University of Genova, Genova, Italy

**Keywords:** Blood-brain barrier model, Polyelectrolyte complex membranes, Tubular structures, Mechanical compression, Endothelial barrier function

## Abstract

The development of physiologically relevant *in vitro* models of the blood-brain barrier (BBB) is critical for reliable assessment of drug permeability and neurotherapeutic transport. Current platforms often fail to reproduce the three-dimensional geometry and mechanical compliance of cerebral microvessels, limiting their translational relevance. Here, we report the fabrication of soft, flexible and self-supporting tubular membranes *via* polyelectrolyte complexation of alginate and ε-poly-L-lysine, yielding cylindrical constructs that closely mimic the architecture and flexibility of small brain vessels. The resulting biomaterials support robust endothelial cell adhesion and the formation of a functional barrier, exhibiting controlled permeability consistent with selective molecular transport. Importantly, the compliant tubular constructs enable the application of external mechanical compression, allowing controlled modulation of vessel deformation and barrier integrity in a manner relevant to pathological conditions such as tumor-induced vascular compression. By integrating physiologically relevant cylindrical geometry with a mechanically compliant and deformable microenvironment, this platform provides a tunable and reproducible basis for endothelial barrier formation and mechanical perturbation, enabling the development of a more biomimetic *in vitro* BBB model.

## Graphical abstract

**Figure:**
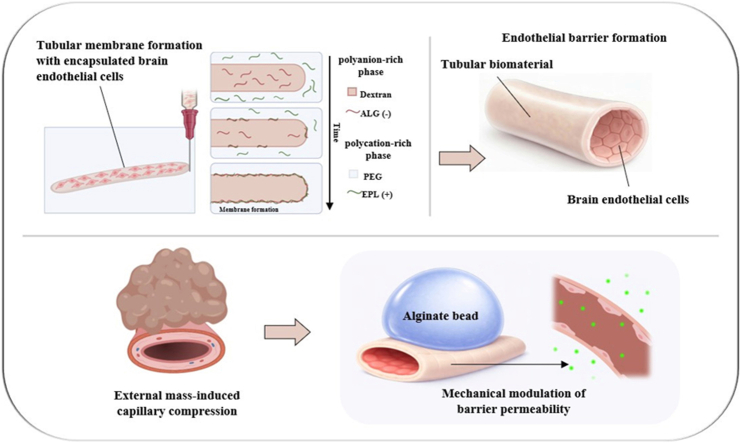

## 1 Introduction

The blood-brain barrier (BBB) is a highly specialized and tightly regulated interface lining the brain's capillaries that controls molecular exchange between the systemic blood circulation and the central nervous system [1,2]. Formed primarily by brain microvascular endothelial cells interconnected by complex tight junctions, the BBB plays a crucial role in maintaining brain homeostasis while restricting and regulating the passage of potentially harmful substances [1,2]. This restrictive nature of the BBB often limits the delivery of therapeutic agents to the brain, posing a major challenge for the treatment of neurological disorders and brain tumors [[1], [2], [3], [4]]. Consequently, physiologically relevant *in vitro* BBB models are essential for advancing our understanding of barrier mechanisms, disease-associated dysfunction, and drug crossing [5,6].

Over the past decades, several *in vitro* BBB models have been developed, ranging from static transwell systems to microfluidic organ-on-chip platforms. While these approaches have provided valuable insights, many fail to capture the key structural and functional features of the native BBB [[5], [6], [7], [8]]. *In vivo*, brain capillaries exhibit a cylindrical geometry, with endothelial cells organized circumferentially around a narrow lumen [2]. This 3D architecture is critical for endothelial polarization, junctional organization, and barrier function, yet it is poorly reproduced in conventional planar models [[5], [6], [7]]. Here, we use a simple self-assembly strategy based on natural biomaterials to generate cytocompatible and flexible tubular constructs. Compared to organ-on-chip systems, which often require complex fabrication processes and specialized equipment, this approach offers a simpler and more cost-effective alternative for the rapid generation of stable membranes with tunable properties. In addition, the resulting soft tubular structures provide a more physiologically relevant mechanical environment compared to conventional rigid substrates such as plastic membranes or polydimethylsiloxane (PDMS) [9,10]. In contrast to planar Transwell systems and rigid microfluidic channels, the platform also provides a cylindrical lumen and supports radial transport across a thin deformable wall, capturing key aspects of the vascular microenvironment [9,10].

Furthermore, many existing BBB platforms mainly focus on biochemical cues while neglecting the physical and mechanical factors that also influence barrier behavior. Mechanical forces are vital regulators of vascular function in both healthy and diseased conditions. In the brain, endothelial cells operate within a physically confined environment where vessel geometry, extracellular matrix (ECM) mechanics, such as stiffness and structural support, blood flow dynamics, and continuous interactions with surrounding cells together define the mechanical signals they experience [11]. Beyond these intrinsic cues, microvessels may also experience external physical perturbations generated by nearby pathological structures. For example, tumor expansion or perivascular protein accumulations such as amyloid deposits can produce growth-induced solid stress that applies localized compression to adjacent capillaries [[11], [12], [13], [14], [15]]. Unlike luminal shear stress, these externally applied loads deform the vessel wall and alter endothelial mechanics, potentially contributing to lumen narrowing and changes in barrier behavior observed *in vivo*. However, the direct causal relationship between controlled capillary compression and endothelial barrier responses has not been systematically quantified. One major limitation in addressing this question is the lack of *in vitro* BBB models allowing controlled mechanical perturbation. Most current BBB platforms are not designed to reproduce mechanical compression, limiting the ability to assess the direct impact of mechanical forces alone [[5], [6], [7], [8]]. There is therefore a clear need for experimental systems that combine a biomimetic vascular geometry with the ability to apply defined mechanical stress and quantitatively assess its impact on BBB integrity and transport behavior [[5], [6], [7], [8],^11^,^12^,^15^].In this work, we present a bioinspired tubular *in vitro* BBB model designed to address these challenges. The platform leverages an ionically assembled polyelectrolyte-complex biomaterial, previously developed and extensively characterized by our group [16,17], in which oppositely charged biopolymers form stable hollow tubular membranes under mild, cell-compatible conditions [18]. In our earlier work [19], we demonstrated high reproducibility and geometric fidelity across fabrication conditions, supported by systematic structural characterization and an AI-driven (Gaussian process-based) framework to capture variability and predict material properties. These features make the system suitable as a scaffold for BBB modeling.

Building on this established biomaterial platform, we engineer a tubular BBB model that supports the formation of a continuous endothelial monolayer lining the inner surface of the tube, closely resembling the cylindrical organization of brain capillaries [2]. A key feature of the model is its intrinsic mechanical flexibility, which enables the application of controlled external compression without altering biochemical culture conditions. Using well-defined alginate beads as compressive elements, localized mechanical stress is applied to endothelialized tubes to emulate key aspects of capillary compression in pathological conditions [15,[20], [21], [22]].

This configuration allows direct comparison between uncompressed (physiological) and mechanically compressed (pathological) BBB states within the same experimental framework. Barrier integrity is evaluated through a combination of permeability measurements, electrical impedance spectroscopy, and drug transport assays, providing complementary functional readouts within a physiologically relevant tubular geometry [[5], [6], [7], [8]].

## 2 Results

**Fig. 1:**
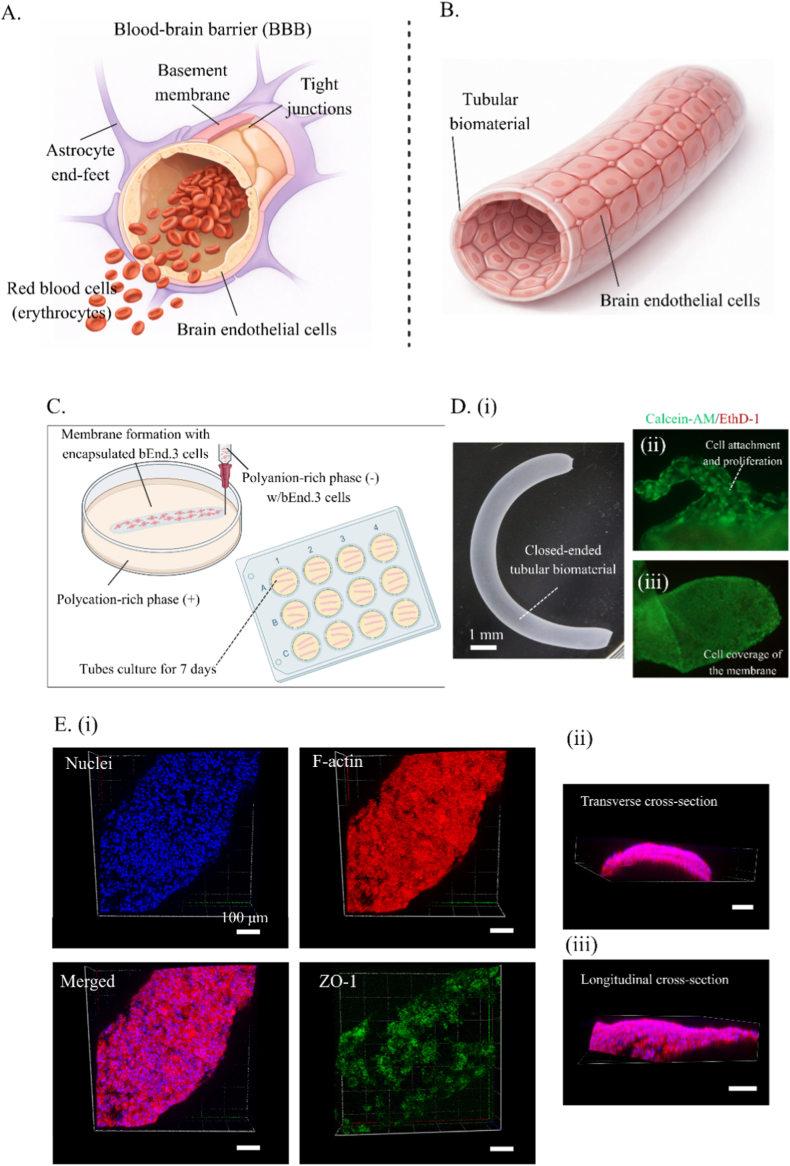
**Fabrication and endothelialization of a tubular BBB model. (A)** Schematic representation of the blood-brain barrier. **(B)** 3D illustration of the tubular and flexible biomaterial lined with brain endothelial cells. **(C)** Fabrication strategy for the tubular biomaterial, based on membrane formation *via* polyelectrolyte complexation, including encapsulation of bEnd.3 cells within the polyanion-rich phase and subsequent culture of the tubular constructs for 7 days to enable endothelial attachment and maturation on the membrane surface. **(D)** Endothelialization of the tubular biomaterial: **(i)** representative image of the flexible, closed-ended tubular construct; **(ii)** live/dead staining (calcein-AM/EthD-1) demonstrating endothelial cell attachment and proliferation; **(iii)** live/dead staining confirming uniform cell coverage of the membrane. **(E)** Structural and cytoskeletal organization of the endothelial layer after 7 days in culture: **(i)** immunofluorescence staining of nuclei, F-actin, and the tight junction protein ZO-1, including merged images; **(ii)** transverse cross-section of the endothelialized tubular construct; **(iii)** longitudinal cross-section of the endothelialized tubular construct.

*In vivo*, brain capillaries consist of a continuous cylindrical monolayer of endothelial cells arranged with circumferential alignment around the lumen [23], which tightly regulates molecular transport between the bloodstream and neural tissue (Fig. 1A). To replicate this architecture *in vitro*, we fabricated a biomimetic tubular model designed to host a BBB endothelial cell layer (Fig. 1B). The fabrication strategy relies on the interfacial complexation of oppositely charged polyelectrolytes, namely sodium alginate (2% w/w) and ε-poly-L-lysine (PLL; 0.75% w/w) [16,17,24]. To stabilize the interface and spatially confine the complexation process, an aqueous two-phase system composed of immiscible polymers, poly (ethylene glycol) 17% w/w and dextran 15% w/w, was used to facilitate membrane formation (Fig. 1C). Murine brain-derived endothelial cells (bEnd.3) were suspended within the alginate-rich phase and subsequently extruded into the PLL-rich phase, triggering rapid and spontaneous formation of a self-supporting tubular membrane with closed edges encapsulating the cells (Fig. 1D (i)). Following fabrication, the tubular constructs were maintained under static culture conditions for up to seven days, a period needed to allow endothelial cell adhesion to the luminal surface, proliferation and progressive development of a confluent monolayer lining the inner wall of the tube (Fig. 1D (ii-iii)).

Macroscopically, the models exhibited uniform geometry and mechanical stability under standard culture conditions (37 °C). The tubes were produced as continuous structures and sectioned into ∼0.5 cm segments, with consistent sizes along their length and an average membrane thickness of ∼8 μm. The inner diameter decreased from ∼1023 μm before endothelialization to ∼597 μm after 7 days, due to cell-mediated compaction (Fig. S1). Immunofluorescence analysis confirmed the establishment of endothelial characteristics along the entire length of the tube, including homogeneous nuclear distribution and a well-organized F-actin cytoskeleton. Notably, continuous expression of the tight junction protein zonula occludens-1 (ZO-1) was observed, indicating the formation of a mature paracellular barrier (Fig. 1E (i)). Orthogonal transverse and longitudinal cross-sectional imaging verified further the presence of a continuous endothelial monolayer conforming to the tubular geometry of the scaffold (Fig. 1E (ii-iii)). Importantly, the highly compliant tubular constructs, previously reported to exhibit Young's modulus values in the range of ∼60–300 kPa, provide a mechanical environment that more closely resembles that of brain capillaries compared to conventional rigid substrates (Fig. S2).

**Fig. 2:**
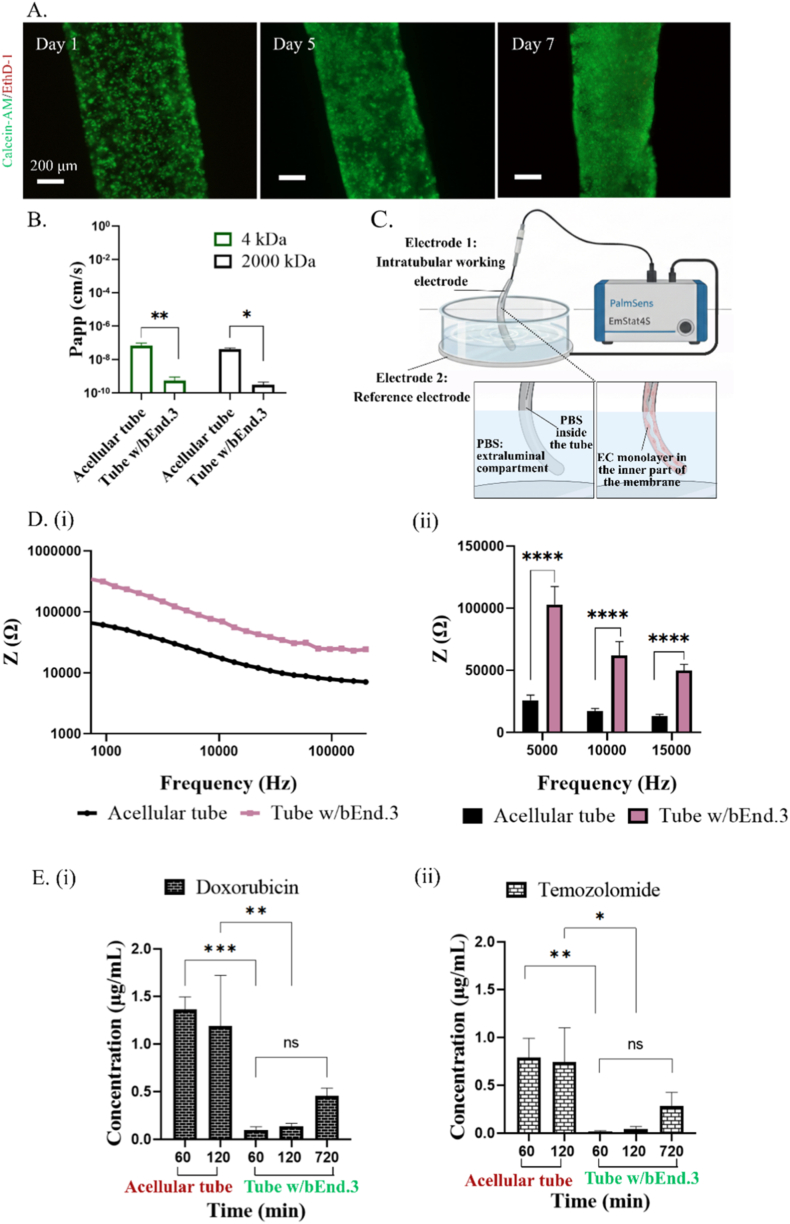
**Functional characterization of the endothelialized tubular BBB model. (A)** Representative live/dead fluorescence images of endothelialized tubular biomaterials at day 1, 5, and 7 in culture, stained with calcein-AM (live cells, green) and ethidium homodimer-1 (EthD-1; dead cells, red), showing progressive endothelial attachment, proliferation, and formation of a confluent monolayer over time. **(B)** Apparent permeability coefficients (*Papp*) of FITC-dextran (4 kDa and 2000 kDa) across acellular control tubes and endothelialized tubes (tube w/bEnd.3), demonstrating significantly reduced permeability upon endothelialization. **(C)** Schematic representation of the experimental setup for impedance measurements. **(D)** Electrical impedance spectroscopy of tubular constructs: **(i)** impedance magnitude (*Z*) as a function of frequency for acellular control and endothelialized tubes; **(ii)** quantification of impedance values at selected frequencies (5, 10, and 15 kHz), showing significantly higher impedance in endothelialized tubes compared to acellular controls, indicative of enhanced barrier integrity. **(E)** Drug permeability assessment across the tubular BBB model: **(i)** doxorubicin and **(ii)** temozolomide concentrations measured in the receiving compartment over time for acellular control and endothelialized tubes, demonstrating restricted transport in the presence of the endothelial barrier. Data are presented as mean ± SD; statistical significance is indicated (∗*p* < 0.05, ∗∗*p* < 0.01, ∗∗∗*p* < 0.001, ∗∗∗∗*p* < 0.0001; ns: not significant). (For interpretation of the references to colour in this figure legend, the reader is referred to the Web version of this article.)

Live/dead staining was performed to assess cell viability and spatiotemporal organization within the tubular constructs. The analysis revealed high viability of bEnd.3 cells throughout the culture period, with minimal evidence of cell death inside the tubular membrane (Fig. 2A). At early timepoints (day 1), endothelial cells appear with a sparse and discontinuous distribution pattern along the luminal surface. Over time, progressive cell adhesion and proliferation were observed, leading to increased surface coverage. By day 7, bEnd.3 cells had already formed a dense and continuous endothelial layer lining the inner surface of the tube, indicating the successful endothelialization and the monolayer maturation (Fig. 2A).

To assess barrier permeability as a functional readout of the model, permeability assays were performed using fluorescein isothiocyanate (FITC)-labeled dextran tracers with molecular weights of 4 kDa and 2000 kDa [[25], [26], [27]]. Apparent permeability coefficients (*Papp*) were quantified for endothelialized tubular constructs and were compared with acellular control tubes. Following tracer exposure for 24 h, quantitative analysis revealed a statistically significant reduction in permeability for bEnd.3-endothelialized tubes compared to acellular controls for both dextran tracers (Fig. 2B). This decrease in *Papp* demonstrates the formation of an effective endothelial barrier capable of restricting paracellular transport. Notably, the decrease in permeability was more pronounced for the 2000 kDa dextran, highlighting the size-selective nature of transport across the endothelialized membrane. Reported BBB permeability values typically range from ∼10^−5^ to 10^−7^ cm/s in conventional *in vitro* models, while tighter BBB-on-chip or iPSC-derived systems may reach ∼10^−7^-10^−8^ cm/s [[28], [29], [30], [31]]. In this context, our tubular models exhibited lower permeability values (∼10^−9^-10^−10^ cm/s for 4 kDa FITC-dextran), indicating a highly restrictive barrier. In contrast, acellular tubes displayed substantially higher permeability, confirming that barrier function is cell-driven. Consistent with BBB physiology, permeability decreased with increasing molecular size, reflecting the size-selective nature of transport across the barrier.

Barrier integrity was further assessed using electrochemical impedance spectroscopy (EIS), which provides a sensitive, non-invasive measure of endothelial tightness (Fig. 2C). Impedance spectra measured as a function of frequency showed a substantial increase in impedance magnitude in bEnd.3-endothelialized tubes with respect to acellular controls. Electrical measurements showed higher impedance values for endothelialized models compared to acellular controls (Fig. 2D (i)). For clarity and quantitative comparison, impedance values were extracted at selected distinct frequencies, chosen to capture barrier-dependent changes (Fig. 2D (ii)). At these frequencies, endothelialized models exhibited markedly higher impedance values, reflecting increased resistance to ionic transport across the barrier. This electrical behavior is indicative of tight intercellular junctions and reduced paracellular leakage, supporting the permeability results.

To evaluate drug transport across the tubular BBB model, temozolomide and doxorubicin were selected as clinically relevant anticancer agents with well-characterized but markedly different abilities to cross the BBB [32]. Temozolomide is a small, moderately lipophilic molecule known to penetrate the BBB and is commonly used in glioblastoma therapy, whereas doxorubicin is a larger, more hydrophilic compound that is mostly excluded from the brain due to limited BBB permeability [[33], [34], [35], [36]]. The use of these two drugs allowed us to test whether the model reflects clinically relevant differences in BBB drug permeability. Endothelialized models and acellular controls were exposed to a mixture of both drugs at a concentration of 10 μg/mL each, and drug translocation across the tubular wall was quantitatively assessed at 1, 2, and 12 h. In both cases, bEnd.3-tubes exhibited a statistically significant reduction in drug passage compared to acellular models (Fig. 2E (i-ii)). While temozolomide and doxorubicin both showed restricted passage at early time points (1 and 2 h), doxorubicin displayed increased translocation at 12 h compared to temozolomide. However, this increase was not statistically significant when compared to earlier time points (Fig. 2E (i-ii)). This observation indicates a time-dependent trend rather than a definitive change in barrier permeability, and suggests that drug-endothelial interactions may influence transport behavior over prolonged exposure without compromising overall barrier integrity. While temozolomide crosses the BBB more efficiently *in vivo* due to its lipophilicity, the more hydrophilic character of doxorubicin likely promotes greater interaction with the alginate/ε-polylysine matrix, contributing to its higher measured accumulation. Importantly, both drugs showed significantly reduced transport after endothelialization, confirming functional barrier formation. Overall, the reduction in drug crossing observed in endothelialized models demonstrates that the presence of a confluent bEnd.3 monolayer imposes an effective barrier to molecular transport, consistent with functional BBB behavior.

**Fig. 3:**
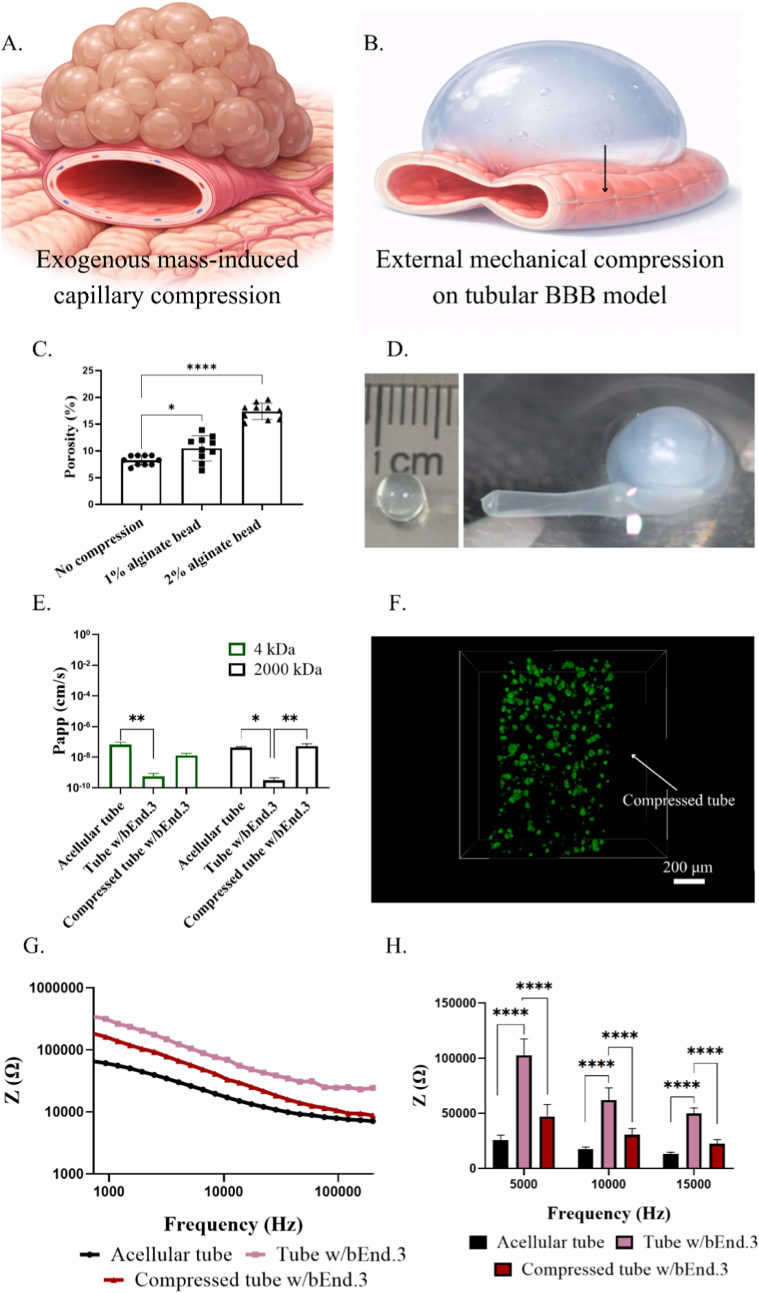
**Localized external loading induces structural and functional remodeling of tubular BBB constructs. (A)** Conceptual illustration of capillary deformation caused by an exogenous space-occupying mass, leading to luminal narrowing *in vivo*. **(B)** Biomimetic in vitro representation of localized external mechanical compression applied to an endothelialized tubular BBB construct, modeling controlled capillary deformation under defined loading conditions. **(C)** Quantification of membrane porosity following bead-induced mechanical compression, showing compression-driven alterations in the porous structure of the tubes as a function of alginate concentration. **(D)** Representative image of a tubular biomaterial subjected to external mechanical compression. **(E)** Apparent permeability coefficients (*Papp*) of FITC-dextran (4 kDa and 2000 kDa) across acellular control tubes, normal endothelialized tubes, and mechanically compressed endothelialized tubes, indicating altered barrier permeability under compression. **(F)** Representative 3D immunofluorescence image showing ZO-1 distribution in an endothelialized tubular construct after mechanical compression. **(G)** Electrical impedance spectroscopy showing impedance magnitude (*Z*) as a function of frequency for normal endothelialized tubes, compressed endothelialized tubes, and acellular tubes. **(H)** Quantification of impedance values at selected frequencies (5, 10, and 15 kHz), demonstrating a significant reduction in barrier integrity upon mechanical compression. Data are presented as mean ± SD; statistical significance is indicated (∗*p* < 0.05, ∗∗*p* < 0.01, ∗∗∗*p* < 0.001, ∗∗∗∗*p* < 0.0001; ns: not significant).

During pathological progression, the BBB undergoes both structural and functional alterations [11,14,15,37]. Beyond biochemical and angiogenic remodeling, space-occupying processes can generate external mechanical stresses that compress surrounding brain microvessels and deform capillary geometry, contributing to altered barrier behavior (Fig. 3A). However, the direct effects of localized mechanical compression on endothelial integrity remain insufficiently defined, largely due to the limited availability of in vitro BBB models that allow controlled and reproducible mechanical perturbation [11,12,20]. Building upon the bioengineered tubular BBB platform described above, we introduced a localized external loading approach to model mechanically induced vascular deformation in a defined and isolated manner. Specifically, bEnd.3-endothelialized tubes were subjected to externally applied static compression using calibrated alginate beads (Fig. 3B), enabling direct investigation of BBB responses to spatially confined external mechanical stress. To establish a controlled and reproducible strategy for applying external mechanical compression, spherical alginate beads fabricated from two different polymer concentrations (1% and 2% w/w sodium alginate) were first evaluated as compressive elements. Beads fabricated with 1% and 2% alginate exhibited comparable macroscopic dimensions but produced distinct mechanical responses upon contact with the tubular constructs. Despite their similar size, increasing alginate concentration led to significantly different effects on the membranes, with 2% alginate beads inducing a marked increase in membrane porosity compared to 1% beads (Fig. 3C). Based on these observations, 2% alginate beads were selected for subsequent compression experiments. External compression was applied by gently positioning 2% alginate beads in direct contact with endothelialized tubes at day 7 of culture for 24 h, generating localized compressive stress on the tubular structure with an estimated applied load of ∼100 Pa (Fig. 3D). The applied load models external solid stress rather than intraluminal pressure, mimicking the compressive forces generated by tumor expansion in surrounding brain tissue. Reported stresses at tumor interfaces are within a similar range (∼20-110 Pa), supporting the physiological relevance of the applied compression. Importantly, the compliant alginate bead enables a soft tissue-like mechanical interaction rather than rigid indentation [20].

This approach produced controlled deformation characterized by localized lumen narrowing. The compliant nature of the tubular scaffold enabled deformation under loading, allowing direct comparison between uncompressed (physiological) and externally compressed (pathological-like) BBB models under otherwise identical culture conditions.

The functional impact of mechanical compression on barrier performance was assessed by permeability measurements. Compressed bEnd.3-endothelialized tubes exhibited an increase in apparent permeability (*Papp*) to both 4 kDa and 2000 kDa FITC-dextran tracers with respect to uncompressed endothelialized tubes (Fig. 3E). Conversely, uncompressed endothelialized tubes maintained low permeability values consistent with the presence of an intact endothelial barrier. To investigate the structural basis of the observed functional impairment, tight junction organization was examined by confocal microscopy. Immunofluorescence staining for the tight junction protein ZO-1 in compressed bEnd.3-endothelialized tubes revealed disrupted junctional organization and reduced continuity along the endothelial layer (Fig. 3F). The altered ZO-1 distribution observed under compression is consistent with mechanically induced perturbation of endothelial junctional architecture and provides a structural correlation to the increased permeability and reduced electrical resistance measured in compressed conditions.

Electrical characterization using electrochemical impedance spectroscopy (EIS) provided complementary evidence of compression-induced barrier disruption. Compressed endothelialized tubes showed a clear reduction in impedance magnitude across the measured frequency range compared to uncompressed tubes (Fig. 3G). Quantitative analysis at selected discrete frequencies confirmed a statistically significant decrease in impedance following compression (Fig. 3H), with values approaching those measured in acellular controls, consistent with compromised barrier resistance.

Collectively, all these data demonstrate that externally applied mechanical compression using 2% alginate beads induces structural and functional alterations in the endothelialized tubular BBB model. The combination of increased permeability, disruption of tight junction organization, and reduction in electrical resistance indicate loss of barrier integrity under sustained mechanical stress, establishing this platform as a controlled *in vitro* system to study compression-induced BBB dysfunction relevant to glioblastoma progression [11].

**Fig. 4:**
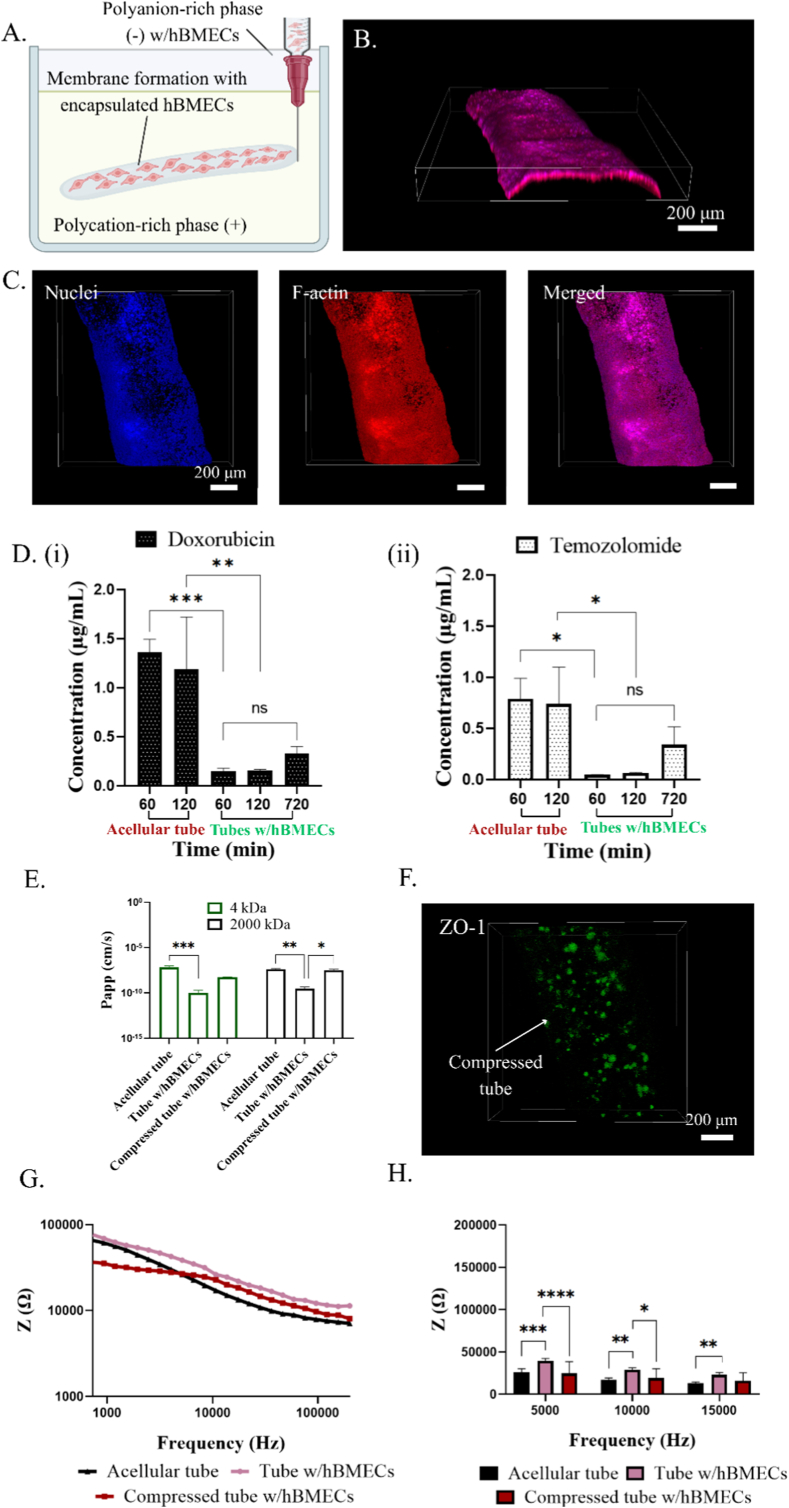
**Fabrication and functional characterization of hBMECs-endothelialized tubular BBB models. (A)** Schematic illustration of tubular biomaterial fabrication *via* polyelectrolyte complexation, including membrane formation with encapsulated human brain microvascular endothelial cells (hBMECs). **(B)** Representative 3D reconstruction of an hBMECs-endothelialized tubular construct. **(C)** Immunofluorescence images showing endothelial organization within the tubular construct after 7 days in culture, including nuclei (blue), F-actin (red), and merged views. **(D)** Drug transport assessment across the tubular BBB model: **(i)** doxorubicin and **(ii)** temozolomide concentrations measured over time for acellular control and hBMECs-endothelialized tubes. **(E)** Apparent permeability coefficients (*Papp*) of FITC–dextran (4 and 2000 kDa) across acellular control tubes, normal endothelialized tubes, and compressed endothelialized tubes. **(F)** Representative 3D immunofluorescence image showing ZO-1 distribution in an endothelialized tubular construct subjected to mechanical compression. **(G)** Electrical impedance spectroscopy showing impedance magnitude (*Z*) as a function of frequency for normal endothelialized tubes, compressed endothelialized tubes, and acellular controls. **(H)** Quantification of impedance values at selected frequencies (5, 10, and 15 kHz), demonstrating reduced barrier integrity upon mechanical compression. Data are presented as mean ± SD; statistical significance is indicated (∗*p* < 0.05, ∗∗*p* < 0.01, ∗∗∗*p* < 0.001, ∗∗∗∗*p* < 0.0001; ns: not significant). (For interpretation of the references to colour in this figure legend, the reader is referred to the Web version of this article.)

Further validation of the tubular BBB platform was performed using human brain microvascular endothelial cells (hBMECs). As schematically illustrated in Fig. 4A, hBMECs were incorporated into the polyanion-rich phase during fabrication, resulting in tubular membranes supporting a human endothelial layer along the luminal surface. 3D imaging confirmed successful endothelialization, with hBMECs uniformly distributed along the tubular structure (Fig. 4B). The human endothelial cells displayed a well-organized architecture within the cylindrical geometry, as evidenced by homogeneous nuclear distribution and a continuous, organized F-actin network aligned with the tube axis (Fig. 4C).

Functional barrier performance was assessed through drug transport studies using temozolomide and doxorubicin (Fig. 4D(i–ii)). In hBMECs-endothelialized tubes, doxorubicin transport was significantly reduced compared to acellular controls at early time points, indicating effective restriction of drug passage by the endothelial barrier. No statistically significant increase in doxorubicin translocation was observed at prolonged incubation times, supporting the maintenance of barrier integrity over the course of the experiment. Temozolomide transport was likewise significantly reduced in endothelialized tubes relative to acellular controls at all tested time points, confirming the presence of a functional human endothelial barrier. Although a higher temozolomide signal was detected at 12 h compared to earlier time points in endothelialized samples, this increase did not reach statistical significance, indicating that barrier function remained preserved over time.

Permeability of the human BBB model was also assessed in both uncompressed (physiological) and mechanically compressed conditions using the same experimental protocol as for the bEnd.3-endothelialized tubes, employing FITC-dextran tracers of 4 and 2000 kDa (Fig. 4E). Both 4 kDa and 2000 kDa FITC-dextran exhibited significantly reduced apparent permeability in hBMEC-endothelialized tubes compared to acellular controls, confirming effective barrier formation (Fig. 4E). Mechanical compression led to a modest increase in permeability relative to uncompressed endothelialized tubes. These results indicate a partial, but not complete, loss of barrier function under mechanical stress.

Structural changes underlying this functional alteration were examined by imaging tight junction organization. Immunofluorescence staining for the tight junction protein ZO-1 revealed disrupted junctional organization and reduced continuity in compressed hBMEC-endothelialized tubes (Fig. 4F). The altered ZO-1 distribution observed under compression provides a structural correlation to the increased permeability measured in mechanically stressed conditions.

The electrical barrier properties were subsequently evaluated using electrochemical impedance spectroscopy (EIS). Uncompressed hBMECs-endothelialized tubes exhibited significantly higher impedance values compared to acellular controls, indicating the formation of an electrically resistive endothelial barrier (Fig. 4G). In contrast, compressed hBMECs-endothelialized tubes showed a reduction in impedance magnitude across the measured frequency range relative to uncompressed tubes. Quantitative analysis at selected distinct frequencies confirmed statistically significant decreases in impedance following compression (Fig. 4H), consistent with increased ionic conductance. Together, these electrical measurements indicate compression-induced impairment of endothelial barrier resistance.

Together, these findings indicate that the tubular BBB model with human endothelial cells reproduces important aspects of BBB drug transport behavior. When considered alongside the structural, permeability, and electrical measurements, the results confirm that the platform can be successfully translated to human brain microvascular endothelial cells. Although increased variability was observed compared to the murine bEnd.3 system, the hBMECs-based model reproducibly exhibited endothelial barrier formation, size-selective permeability, and mechanically induced barrier dysfunction. In conclusion, these features support the relevance of the platform for investigating human BBB physiology and pathology under both physiological and mechanically stressed conditions.

## 3 Materials and methods

### 3.1 Materials

Sodium alginate sourced from brown algae (Mw 120,000–190,000 g mol^−1^) and dextran from *Leuconostoc* spp. (Mw 450,000–650,000 Da) were purchased from Sigma-Aldrich and used as constituents of the polyanion-rich solution. Poly(ethylene glycol; PEG, Mw 8000 Da) was also obtained from Sigma-Aldrich. ε-Poly-L-lysine (ε-PLL; Epolyly Pure, Mw ≈ 4700 g mol^−1^), produced *via* fermentation of *Streptomyces albulus* PD-1, was provided by Handary S.A. and used as a constituent of the polycation-rich solution. Phosphate-buffered saline (PBS) was purchased from EuroClone and used for washing and dilution steps. Fluorescein 5(6)-isothiocyanate (FITC, ≥90% purity, HPLC grade) and fluorescein isothiocyanate-dextran (FITC-dextran; Mw 4 and 2000 kDa) were obtained from Sigma-Aldrich and used for structural labeling and permeability assays. For cell adhesion, GRGDSP-functionalized sodium alginate (NOVATACH™ MVG GRGDSP, high molecular weight) was purchased from NovaMatrix. Murine brain endothelial cells (bEnd.3, ATCC® CRL-2299™) were obtained from the American Type Culture Collection (ATCC). Immortalized human brain microvascular endothelial cells (IM-hBMECs) were purchased from Innoprot (Derio, Spain). Paraformaldehyde (PFA), Triton X-100, phalloidin (tetramethylrhodamine-conjugated), Hoechst nuclear stain, and trypsin-EDTA (0.05%) were purchased from Sigma-Aldrich and used for cell fixation, permeabilization, and staining. Tight junctions were labeled using an Alexa Fluor™ Plus 488-conjugated recombinant mouse monoclonal anti-ZO-1 antibody (ZO1-1A12; Thermo Fisher Scientific). Temozolomide was purchased from TCI Chemicals, and doxorubicin hydrochloride was purchased from Sigma-Aldrich, and both were used in drug transport studies. For fabrication of alginate beads used as compressive elements, sodium alginate from brown algae (Mw 120,000–190,000 g mol^−1^; Sigma-Aldrich) and calcium chloride anhydrous (Sigma-Aldrich) were used.

### 3.2 Fabrication and endothelialization of tubular BBB constructs

Endothelial cell suspensions were prepared by trypsinization followed by resuspension at a density of 50·10^6^ cells mL^−1^. The cells were encapsulated within the dispersed phase (polyanion-rich phase), consisting of sodium alginate (2% w/w) dissolved in a dextran solution (15% w/w). To promote endothelial cell adhesion, GRGDSP-functionalized sodium alginate (RGD-modified alginate) was added to polyanion-rich phase at a concentration of 0.5% w/w. Polycation-rich phase consisted of ε-poly-L-lysine (ε-PLL, 0.75% w/w) dissolved in a PEG aqueous solution (17% w/w) and served as the continuous phase for tubular membrane formation. The pH of this phase was adjusted to 7.2 before cell incorporation to ensure physiological conditions. All solutions used for structure fabrication were prepared using PBS with calcium and magnesium ions (Ca^2+^/Mg^2+^) and were sterilized prior to use. Aqueous solutions containing the dissolved polymers (PEG and dextran) were sterilized by filtration through sterile 0.2 μm pore-size filters. Sodium alginate and ε-PLL reagents were sterilized by exposure to ultraviolet (UV) light for 40 min, while ALG-RGD was sterilized by UV exposure for 20 min. Sterilized polyelectrolytes were subsequently added to the filtered polymer solutions under sterile conditions and dissolved using autoclaved magnetic stir bars.

Hollow constructs were fabricated by manually injecting the cell-laden polyanion-rich phase into the polycation-rich phase using a syringe fitted with a 25G needle nozzle. Upon contact between the two phases, spontaneous ionic complexation between the oppositely charged polyelectrolytes led to the formation of a tubular membrane. After approximately 2 min of complexation, the fabricated biomaterials were gently washed three times with PBS containing Ca^2+^ and Mg^2+^ to remove residual phase components, then transferred to fresh PBS and subsequently to the appropriate endothelial cell culture medium. The endothelialized constructs were maintained under standard cell culture conditions at 37 °C in a humidified atmosphere with 5% CO_2_. Culture medium was replaced every 48 h, and constructs were maintained for up to 7 days to allow endothelial cell attachment, spreading, and formation of a confluent monolayer along the luminal surface.

### 3.3 Young's modulus characterization of the tubular membranes

Tubular membrane samples were mounted on a custom support and fixed at the edges using adhesive material, leaving the central membrane region exposed for measurements. Samples were maintained in an aqueous environment (PBS) throughout the analysis to preserve their mechanical properties and prevent dehydration. Force spectroscopy measurements were performed using a NanoWizard IV AFM system (Bruker). Prior to AFM measurements, the regions of interest were identified using a BioMAT Workstation (Bruker, USA), whose portable shuttle-stage design enables accurate relocation and investigation of selected areas on opaque samples.

Silicon nitride triangular cantilevers (DNP-10, Bruker, USA), with a nominal spring constant of 0.24 N/m and a nominal tip radius of curvature of 20 nm, were used for all experiments. Force–distance (FD) curve maps were acquired in QI mode (Bruker, USA) over three independent areas of 15 μm × 15 μm for each sample. Each map consisted of 64 × 64 FD curves, corresponding to a total of 12.288 force curves acquired per sample. The maximum force applied was 7 nN and the tip displacement velocity 15 μm/s. Four independent samples were analyzed.

Young's modulus values were extracted by fitting the approach portion of the FD curves using the Bilodeau approximation of the Hertz–Sneddon contact model, which extends the classical Hertzian formulation to a quadratic pyramidal indenter geometry.

### 3.4 Cell viability, morphology, and immunofluorescence analysis

Cell viability, attachment to the tubular membrane, and proliferation were assessed at predetermined time points (days 1, 5, and 7) using a Live/Dead fluorescence assay. At each time point, endothelialized tubular constructs were incubated in complete culture medium containing Calcein-AM and ethidium homodimer-1 (EthD-1) at final concentrations of 2 μL mL^−1^ and 1 μL mL^−1^, respectively, for 10 min at 37 °C. Following staining, constructs were gently washed with culture medium and imaged using an upright widefield fluorescence microscope (Axio Imager M2, Carl Zeiss).

After 7 days of culture, when a confluent endothelial monolayer had formed, constructs were fixed and stained to visualize cellular distribution within the tubular geometry and to qualitatively assess cell morphology and cytoskeletal organization. Samples were fixed with 4% PFA at 4 °C for 10 min and rinsed twice with PBS. Cell membranes were permeabilized using 0.1% Triton X-100 in PBS for 5 min at room temperature, followed by two PBS washes. For cytoskeletal and nuclear staining, samples were incubated for 30 min at 37 °C in PBS containing 10% goat serum, tetramethyl rhodamine (TRITC)-conjugated phalloidin (2 μg mL^−1^), and Hoechst nuclear stain (5 μg mL^−1^). After staining, constructs were rinsed twice with PBS and imaged using a confocal laser scanning microscope. Confocal fluorescence imaging was performed using a confocal microscope (LSM 900, Carl Zeiss, Germany) and a confocal microscope (C2plus, Nikon, Japan), with voxel sizes of 0.624 × 0.624 × 1.740 μm and 1.24 × 1.24 × 7.1 μm (*X* × *Y* × *Z*) for high-resolution and large field-of-view imaging, respectively.

To assess tight junction maturation in endothelialized constructs, immunofluorescence staining for the tight junction protein zonula occludens-1 (ZO-1) was performed. Samples were fixed in 4% PFA in PBS at 4 °C for 10 min, rinsed twice with PBS, and permeabilized with 0.1% Triton X-100 in PBS for 5 min at room temperature. Non-specific binding was blocked by incubation in a 10% goat serum solution in PBS for 40 min. Samples were then incubated overnight at 4 °C with Alexa Fluor 488–conjugated anti-ZO-1 antibody at a final concentration of 5 μg mL^−1^ in blocking solution. Following antibody incubation, samples were rinsed twice with blocking buffer and counterstained for 30 min at 37 °C with a solution containing Hoechst (5 μg mL^−1^) and TRITC-phalloidin (2 μg mL^−1^). Samples were finally rinsed twice with PBS and imaged by confocal laser scanning microscopy.

### 3.5 Permeability assessment

Barrier permeability was quantified using fluorescein isothiocyanate (FITC)-labeled dextran tracers of two molecular weights (4 and 2000 kDa). Endothelialized tubular constructs were evaluated alongside acellular tubular constructs as controls. All tubes were fabricated and maintained with closed ends to ensure that tracer exchange occurred exclusively through the tubular wall. For the assay, tubular constructs were incubated for 24 h in culture medium containing FITC-dextran (4 kDa at 5 μg/mL or 2000 kDa at 25 μg/mL). Following incubation, constructs were transferred to PBS-filled microcentrifuge tubes and washed thoroughly to remove residual tracer from the external surface. Tubes were then sectioned into two pieces to promote release of tracer retained within the construct. The resulting solution containing released FITC-dextran was collected and fluorescence intensity was quantified using a microplate reader (calibration curves in Fig. S3). Apparent permeability coefficients (*Papp*) were calculated from fluorescence measurements, and values were compared between endothelialized and acellular conditions.

Apparent permeability (*Papp*) of FITC-dextran across the tubular wall was calculated using tube geometry and fluorescence-based tracer quantification. Tube length (*L*) and inner diameter (*d*) were measured, and the inner radius was defined as r=d/2. The luminal surface area available for transport (*A*) and the lumen volume (*V_in*) were calculated as *A = 2πrL* and $V_{in}=\pi r^{2}L$, respectively. After 24 h incubation with FITC-dextran, fluorescence intensity was measured and converted to tracer concentration using a calibration curve. Apparent permeability was then calculated as indicated in Equation (1):

Equation 1 $$P_{app}=\frac{V_{in}}{A\;t}\cdot \frac{C_{rec}}{C_{donor}}$$

where t is the incubation time (s), $C_{donor}$ is the initial tracer concentration, and $C_{rec}$ is the concentration recovered after incubation. When fluorescence intensity values expressed as relative fluorescence units (RFU) were used directly, the concentration ratio was replaced by the corresponding RFU ratio. Apparent permeability values are reported in cm·s^−1^.

### 3.6 Drug transport assay

Drug transport across the tubular BBB constructs was evaluated using temozolomide and doxorubicin. Endothelialized tubes and acellular control tubes were incubated with a cocktail containing both drugs at a final concentration of 10 μg mL^−1^ each, prepared in culture medium. For each experimental condition, three independent tubular constructs were analyzed. After drug treatment, samples were maintained at 37 °C under standard culture conditions, and drug transport was assessed at predefined time points of 1, 2, and 12 h. At each time point, tubular constructs were transferred to a well plate, and the external incubation solution was carefully removed. The tubes were then gently washed twice with PBS to eliminate residual drug from the outer surface and prevent contamination of luminal measurements. The tube was then sectioned at its midpoint to facilitate release of the luminal contents. The recovered solution was collected, transferred to vials, and quantified by high-performance liquid chromatography (HPLC) using a single analytical run designed to resolve both compounds sequentially. Chromatographic separation was initiated under conditions optimized for temozolomide analysis, using a mobile phase consisting of methanol and water containing 1% (v/v) HPLC-grade acetic acid, with a final composition of 25% methanol, 74% water, and 1% acetic acid. Isocratic elution was performed at a flow rate of 0.5 mL min^−1^, and absorbance was monitored at 328 nm. Subsequently, the chromatographic conditions were adjusted to those optimized for doxorubicin analysis, using a mobile phase composed of acetonitrile and water containing 1% (v/v) HPLC-grade acetic acid, with a final composition of 30% acetonitrile, 69% water, and 1% acetic acid. Isocratic elution was performed at a flow rate of 0.8 mL min^−1^, with absorbance monitored at 485 nm. The total run time for the combined analysis was approximately 25 min, allowing complete elution and baseline separation of both compounds. Calibration curves were generated for each drug using known concentrations (0-10 μg mL^−1^) prepared in PBS, and a linear relation between peak area and drug concentration was confirmed (Fig. S4). When samples contained both drugs, chromatographic separation enabled independent quantification of temozolomide and doxorubicin within the same run. Drug transport was expressed as the amount of drug recovered from the tubular lumen at each time point and compared between endothelialized and acellular control tubes.

### 3.7 Electrochemical impedance spectroscopy

Barrier integrity and maturation of the tubular BBB constructs were evaluated using electrochemical impedance spectroscopy (EIS). Measurements were performed using an EmStat4S potentiostat (PalmSens) in a two-electrode configuration. For each measurement, tubular constructs were prepared by cutting one end of the tube to allow electrode access. The tube was positioned vertically in a well filled with PBS without calcium and magnesium. One electrode was placed at the bottom of the well in contact with the external solution, while a second electrode was inserted into the open end of the tube, positioned within the lumen and maintained outside the bulk liquid. This configuration enabled impedance measurements across the tubular wall.

Impedance spectra were acquired using PSTrace 5 software (PalmSens) by applying a sinusoidal AC voltage signal with an amplitude of 10 mV over a frequency range of 1 Hz to 200 kHz. Measurements were performed on normal endothelialized tubes, mechanically compressed endothelialized tubes, and acellular control models to evaluate the contribution of the endothelial layer and the effects of mechanical stress on barrier integrity. Impedance data were analyzed to compare relative changes in barrier resistance between conditions and to assess endothelial maturation and functional integrity.

### 3.8 Alginate bead fabrication and mechanical compression

Alginate beads used as external compressive elements were fabricated from sodium alginate solutions prepared at two different polymer concentrations (1 and 2% w/w). Sodium alginate was dissolved in ultrapure water under gentle stirring until complete dissolution. Alginate beads were fabricated by dispensing defined volumes of alginate solutions dropwise into a 0.1 M calcium chloride bath for 30 min at room temperature to induce ionic crosslinking. Following complete gelation, beads were extensively rinsed with deionized water to remove residual calcium chloride. Sodium alginate and calcium chloride reagents were sterilized by ultraviolet (UV) irradiation for 40 min and subsequently dissolved in sterile ultrapure water using autoclaved magnetic stir bars.

To evaluate membrane deformation, acellular tubular constructs were subjected to external compression using alginate beads. Individual beads were gently positioned in direct contact with the tubular membrane and maintained in place for 24 h under standard culture conditions. Following compression, tubular membranes were rinsed with PBS and stained with fluorescein 5(6)-isothiocyanate (FITC) to visualize membrane morphology. FITC staining was performed by incubating the constructs in FITC solution for 24 h at 4 °C, followed by thorough washing with PBS to remove unbound dye. Membrane deformation and structural features were subsequently examined using confocal laser scanning microscopy with *Z*-stack acquisition, and 3D reconstructions were analyzed using ImageJ software.

### 3.9 Compression of endothelialized tubular constructs

For studies involving endothelialized tubes, compression experiments were performed after completion of endothelial maturation. Tubular constructs seeded with endothelial cells were cultured under standard conditions for 7 days to allow formation of a confluent endothelial monolayer. On day 7, endothelialized membranes were subjected to localized static compression (∼100 Pa) using 2% w/w alginate beads, generating controlled external loading within the confined culture geometry. Constructs were maintained under compression for 24 h at 37 °C in a humidified atmosphere with 5% CO_2_. Following the compression period, endothelialized tubes were either processed for functional assays or fixed for imaging analyses, as described in the corresponding sections. Uncompressed endothelialized tubes cultured under identical conditions were used as physiological controls. This approach enabled direct comparison between uncompressed and mechanically compressed BBB models while maintaining identical biochemical and culture conditions.

### 3.10 Statistical analysis

Data are presented as mean ± SD (*n* = 3). For HPLC analysis, individual replicates were excluded only in cases of clear technical failure (*e.g.*, abnormal peak shape, integration errors, or instrument instability) based on predefined quality-control criteria. In such cases, the corresponding datasets are reported as *n* = 2. Statistical analyses were performed using GraphPad Prism 8. Differences among groups were assessed by two-way ANOVA followed by Tukey's multiple-comparisons test, with *p* < 0.05 considered statistically significant.

## 4 Conclusion

In this study, we present a bioinspired tubular *in vitro* BBB model that integrates physiologically relevant vascular geometry, functional endothelialization of the tube, and controlled mechanical stimuli. Using ionically assembled structures formed through the complexation of oppositely charged polyelectrolytes, sodium alginate and ε-poly-L-lysine, we fabricate stable and flexible tubular constructs that support the formation of a continuous endothelial monolayer, enabling quantitative assessment of barrier integrity through structural imaging, permeability measurements, and electrical impedance analysis.

We demonstrate that the tubular architecture promotes robust BBB function, as evidenced by organized tight junction expression, low macromolecular permeability, and elevated electrical resistance with respect to non-endothelialized control models. Drug transport studies using temozolomide and doxorubicin further supported the functional relevance of the model, revealing differential BBB permeability and stable barrier performance over time. Importantly, these results highlight that drug transport across the engineered barrier is governed not only by molecular size, but also by endothelial-drug interactions, reflecting key aspects of *in vivo* BBB behavior.

A central advantage of this platform is its ability to incorporate controlled external mechanical compression, enabling investigation of mechanically induced vascular deformation associated with pathological external loading. Application of localized compression using well-defined alginate beads resulted in deformation of the tubes and led to increased permeability, disrupted tight junction organization, and reduced electrical resistance. These coordinated functional and structural changes indicate that localized mechanical stress alone can modulate BBB integrity, supporting a mechanistic role for externally imposed vascular compression in barrier dysfunction across mechanically driven pathologies. Crucially, the platform was successfully translated to human brain microvascular endothelial cells. Despite the inherent variability of these cells, the humanized model exhibited endothelial barrier formation, size-selective permeability, and compression-induced dysfunction consistent with observations in the murine system. This cross-species validation underscores the robustness and translational relevance of the approach.

Collectively, this work presents an *in vitro* BBB platform that integrates biomimetic tubular geometry with controlled mechanical deformation, addressing key limitations of conventional planar models. By enabling endothelial barrier formation, assessment of molecular and electrical transport properties, and controlled application of mechanical deformation within a single system, the platform provides a structured framework to examine BBB behavior under defined conditions. Although not aiming to fully recapitulate the complexity of the *in vivo* brain microvasculature, this approach allows systematic investigation of how vascular geometry and mechanically induced deformation influence barrier integrity and transport. More broadly, the platform may support future studies into the mechanobiological aspects of BBB dysfunction and contribute to improved evaluation of drug delivery challenges in the context of central nervous system disease.

## CRediT authorship contribution statement

**Maria Alexaki:** Data curation, Formal analysis, Investigation, Methodology, Validation, Visualization, Writing – original draft. **Attilio Marino:** Data curation, Methodology, Supervision, Writing – review & editing. **Marie Celine Lefevre:** Data curation, Formal analysis, Investigation, Methodology, Writing – review & editing. **Claudio Canale:** Data curation, Investigation, Methodology, Resources. **Davide Odino:** Data curation, Investigation, Methodology. **João F. Mano:** Conceptualization, Supervision, Writing – review & editing. **Mariana B. Oliveira:** Conceptualization, Project administration, Resources, Supervision, Writing – review & editing. **Gianni Ciofani:** Conceptualization, Project administration, Resources, Supervision, Writing – review & editing.

## Declaration of competing interest

The authors declare that they have no known competing financial interests or personal relationships that could have appeared to influence the work reported in this paper.

## Data Availability

Data Avalaible on Zenodo at 10.5281/zenodo.18663081.
