# Supplementary material for: A blood-brain barrier model based on flexible tubes to tailor the biophysical and chemical environment for drug delivery testing
Source: Mater Today Bio. 2026 Jun 2;38:103307. doi: 10.1016/j.mtbio.2026.103307 (PMC13255079; doi:10.1016/j.mtbio.2026.103307)
Supplement: Multimedia component 1 [file mmc1.docx]

Supporting Information for

**A blood-brain barrier model based on flexible tubes to tailor the biophysical and chemical environment for drug delivery testing**

Maria Alexaki^1,2,3*^, Attilio Marino^1^, Marie Celine Lefevre^1^, Claudio Canale^4^, Davide Odino^4^, João F. Mano^3^, Mariana B. Oliveira^2*^, Gianni Ciofani^1*^

^1^Smart Bio-Interfaces, Istituto Italiano di Tecnologia, Pontedera, Italy

^2^The Biorobotics Institute, Scuola Superiore Sant’Anna, Pontedera, Italy

^3^Department of Chemistry, CICECO-Aveiro Institute of Materials, University of Aveiro, Aveiro, Portugal

^4^Physics Department, University of Genova, Genova, Italy


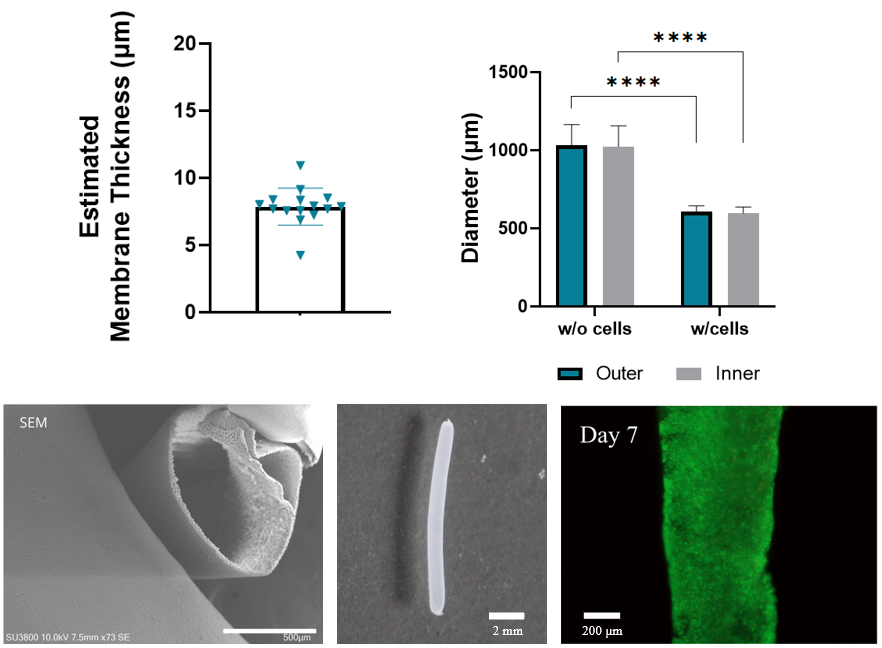


**Figure S1**. Characterization of the hollow tubular membrane construct. Estimated membrane thickness and comparison of outer and inner diameters before and after cell seeding are shown. Representative SEM image of the hollow membrane cross-section, macroscopic image of the tubular construct without cells, and fluorescence image of the cell-seeded construct after 7 days of culture (**** *p* < 0.0001).


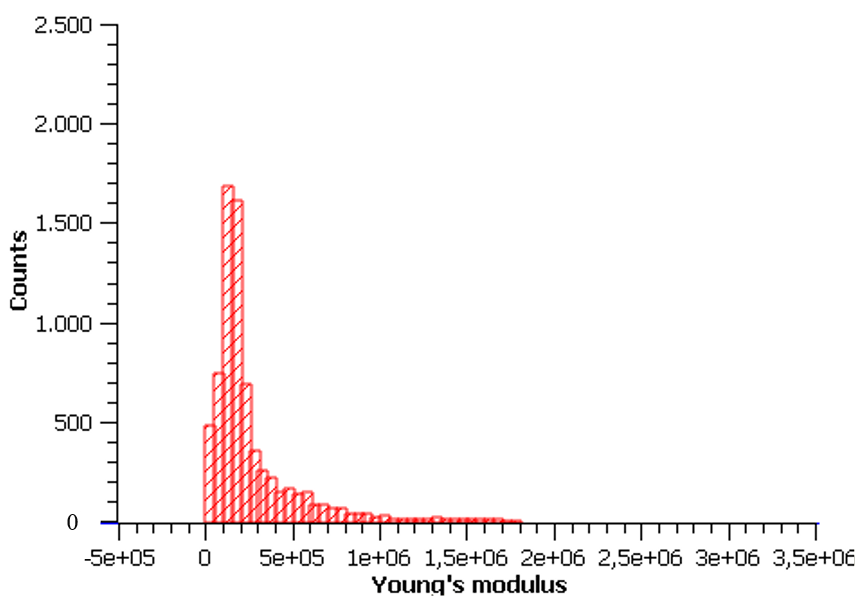


**Figure S2.** Histogram of Young’s modulus values measured by AFM nanoindentation, showing the compliant mechanical profile of the tubular constructs.


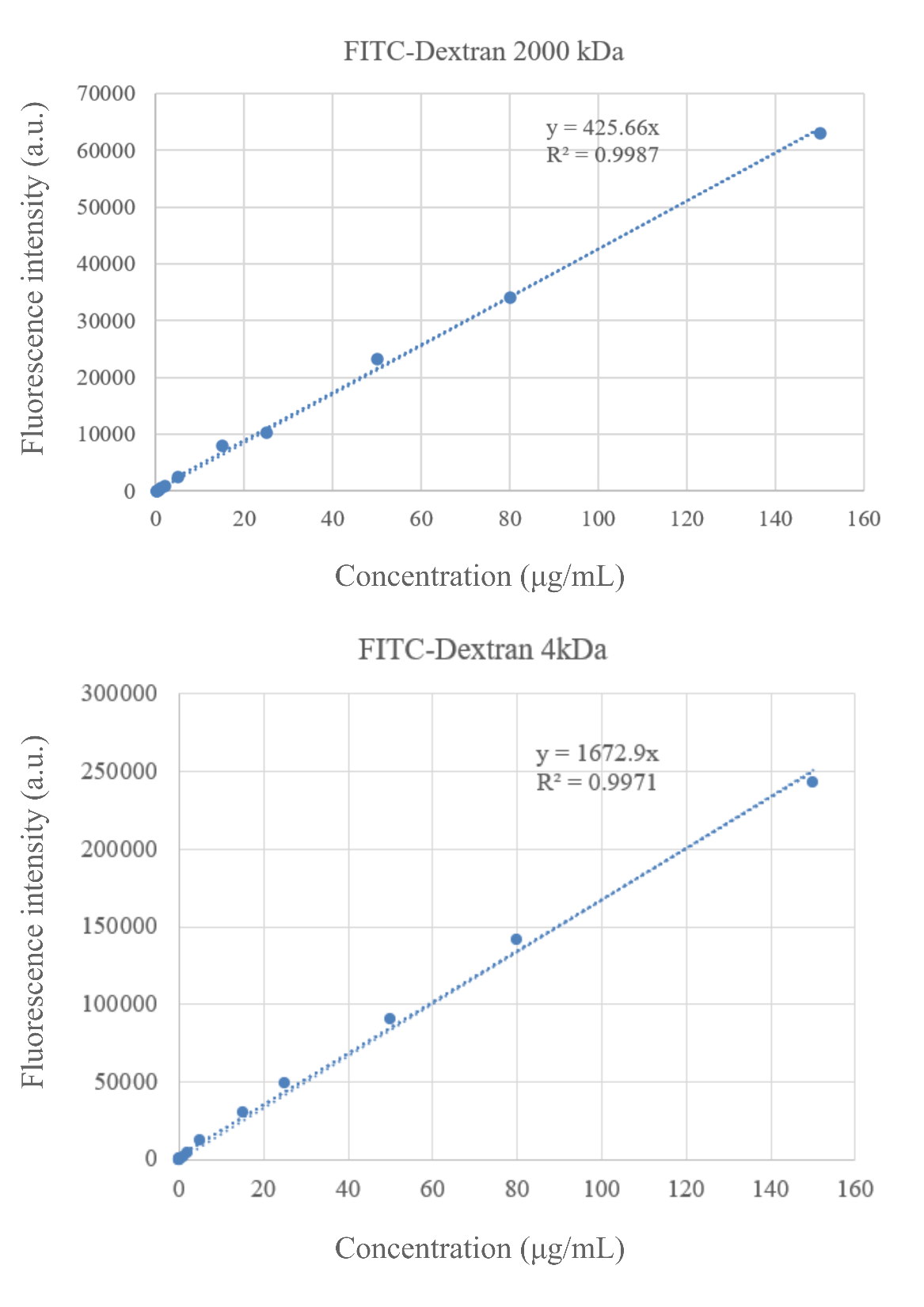


**Figure S3.** Fluorescence calibration curves of FITC-Dextran with molecular weights of 4 kDa and 2000 kDa. Corrected fluorescence intensity (a.u.) is plotted against concentration (µg/mL) after background subtraction. Linear fits and *R²* values are reported.


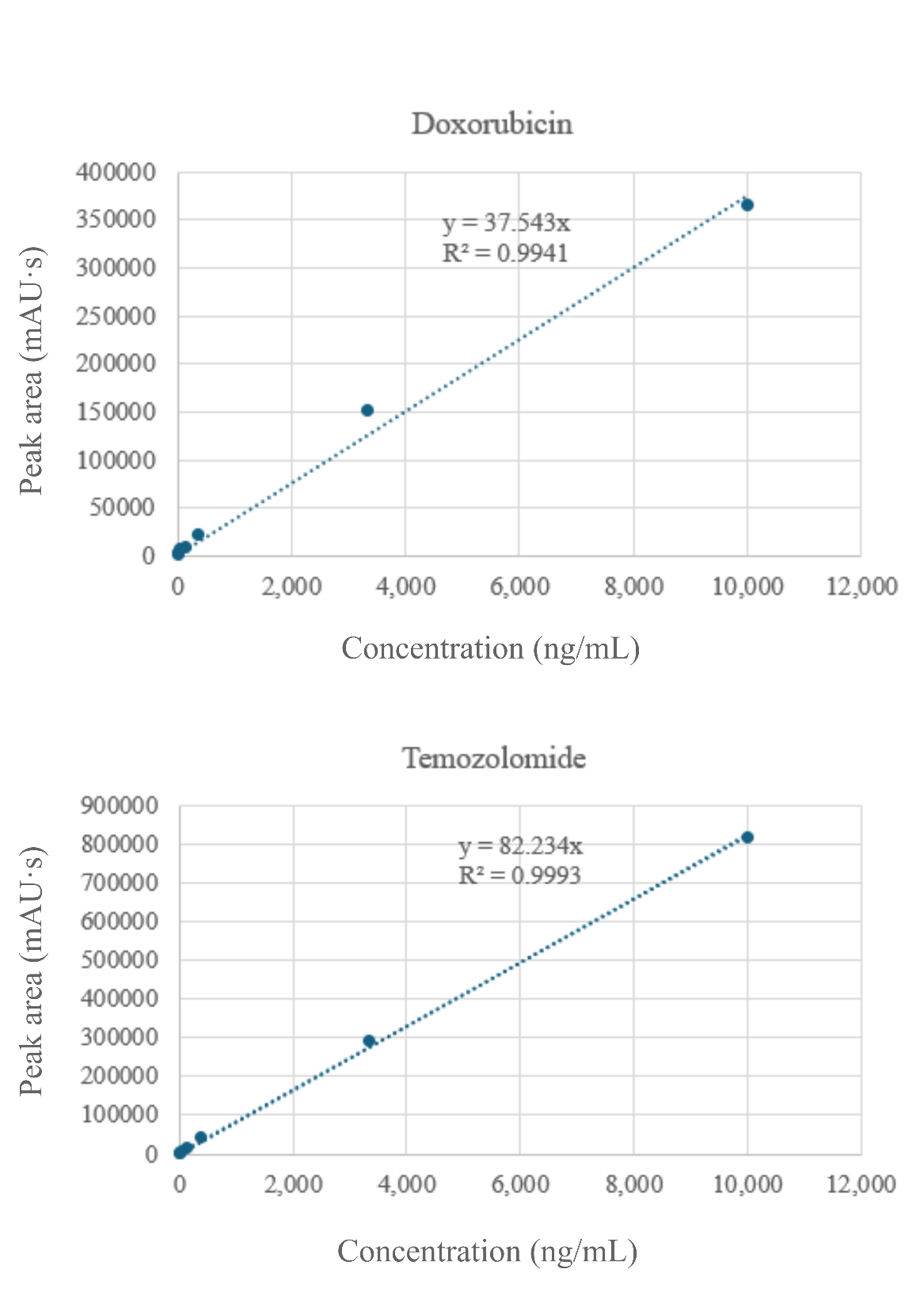


**Figure S4.** HPLC calibration curves for doxorubicin and temozolomide. Peak area (mAU·s) is plotted as a function of analyte concentration (ng/mL). Linear regression equations and coefficients of determination (*R²*) are shown in each panel.
